# Changes in Ugandan Climate Rainfall at the Village and Forest Level

**DOI:** 10.1038/s41598-018-21427-5

**Published:** 2018-02-23

**Authors:** Paddy Ssentongo, Abraham J. B. Muwanguzi, Uri Eden, Timothy Sauer, George Bwanga, Geoffrey Kateregga, Lawrence Aribo, Moses Ojara, Wilberforce Kisamba Mugerwa, Steven J. Schiff

**Affiliations:** 1 Department of Engineering Science and Mechanics, Center for Neural Engineering, PA University Park, USA; 2 Departments of Neurosurgery and Physics, The Pennsylvania State University, University Park, PA University Park, USA; 3 National Planning Authority, Kampala, Uganda; 4 Department of Mathematics and Statistics, Boston University, Boston, USA; 5 Department of Mathematics, George Mason University, Fairfax, VA USA; 6 Map Uganda, Kampala, Uganda; 7 Ugandan National Meteorological Authority, Kampala, Uganda

## Abstract

In 2013, the US National Oceanographic and Atmospheric Administration (NOAA) refined the historical rainfall estimates over the African Continent and produced the African Rainfall Climate version 2.0 (ARC2) estimator. ARC2 offers a nearly complete record of daily rainfall estimates since 1983 at 0.1° × 0.1° resolution. Despite short-term anomalies, we identify an overall decrease in average rainfall of about 12% during the past 34 years in Uganda. Spatiotemporally, these decreases are greatest in agricultural regions of central and western Uganda, but similar rainfall decreases are also reflected in the gorilla habitat within the Bwindi Forest in Southwest Uganda. The findings carry significant implications for agriculture production, food security, wildlife habitat, and economic impact at the community and societal level.

## Introduction

Africa is both the driest and hottest of continents, and its available water is essential for almost all human activities and to support ecological biodiversity^1^. The climate variability of rainfall over East Africa is complex and has been the subject of intense investigation^2^. In 2013, the US National Oceanographic and Atmospheric Administration (NOAA) refined the historical satellite-based rainfall estimates over the African Continent and produced the African Rainfall Climate version 2.0 (ARC2) estimator^3^. This estimator combines daily, geostationary rainfall estimation through infrared cloud reflectivity with ground based rainfall measurements at a fine grid scale at 0.1° x 0.1° resolution (approximately 11 × 11 km at the Equator). The record reaches back to 1983, and continues with real-time daily data production. This paper reports an analysis of the stationarity and climate patterns of rainfall affecting Uganda over the period 1983–2016.

There are many critical uses of such data at such fine temporal and spatial scales. From an economic planning perspective, major construction projects can be mapped to the mean and variability of rainfall for a given location or extent, and engineered to account for anticipated extreme events. Additionally, agricultural planning can be adapted to patterns and predicted changes in rainfall. Moreover, since in a country like Uganda over 70% of the population depends on rain-fed agriculture for food and income^4^, these data may be used to understand population growth and density in given regions and therefore facilitate planning for settlements and food security. From an ecological perspective, habitat limitations for endangered species may place their survival at greater risk.

Furthermore, many human infectious diseases have strong relationships to rainfall (e.g. cholera^5^, malaria^6^, leptospirosis^7^, melioidosis^8^, and the seasonal Neisseria meningitis within the African meningitis belt^9^). Infant infections leading to postinfectious hydrocephalus^10,11^ have been noted to have a significant relationship to rainfall^12^ as well.

**Figure 1:**
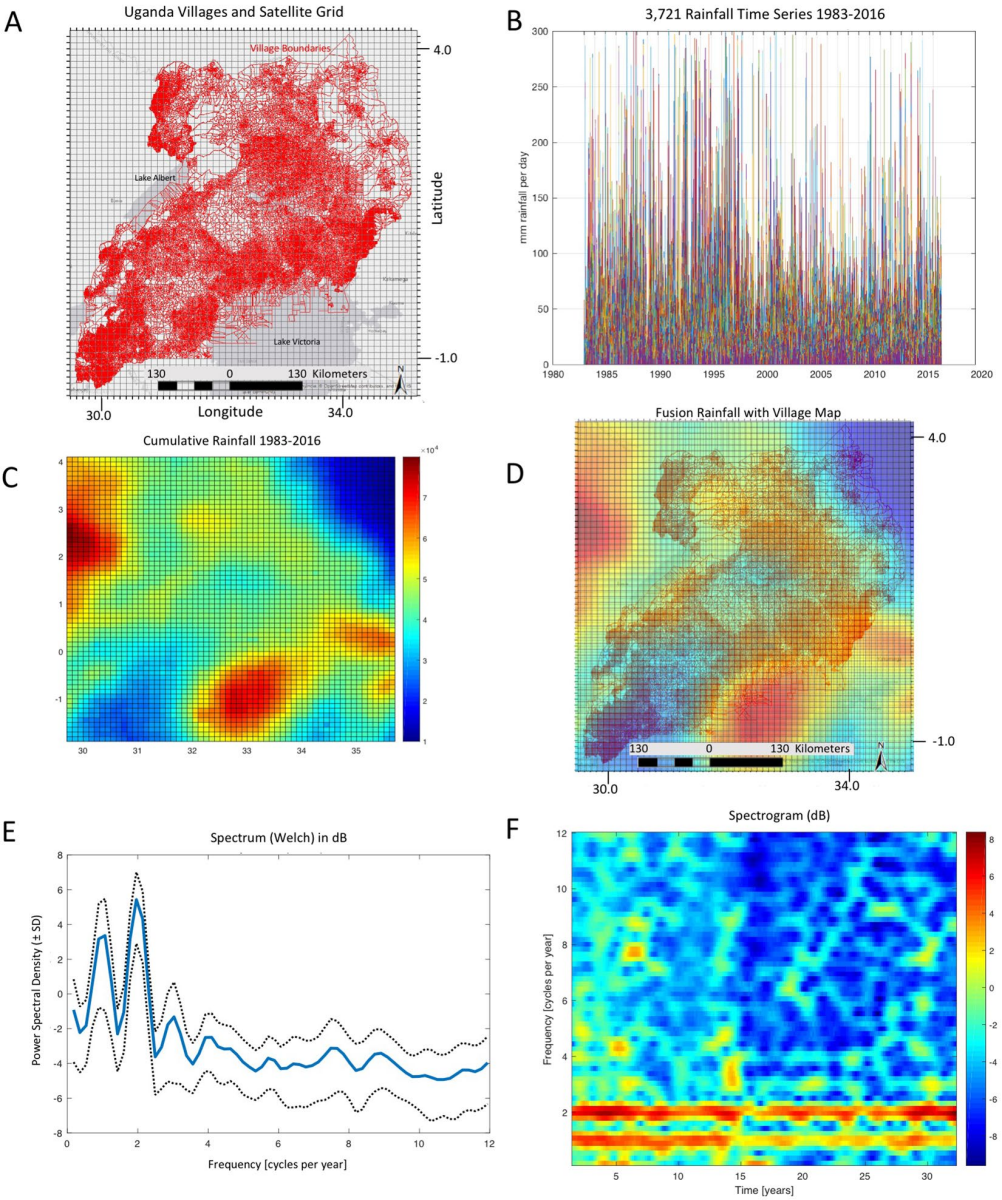
Fusion of satellite rainfall data with map of Uganda at the village level. Shown are the pattern of cumulative rainfall over the 34-year dataset, and the frequency content of the rainfall demonstrating twice-yearly rainy seasons. (**A**) Satellite grid overlay over Uganda showing the 44,034 villages as polygons, overlain with the 61 × 61, 0.1° × 0.1°, satellite grid (map created using ArcGIS 10.4.1, http://support.esri.com/Products/Desktop/ArcGIS-desktop/arcmap/10-4-1). Darker grey regions represent water bodies (Lake Albert upper left, and Lake Victoria lower right). (**B**) Rainfall per day from each of 3,721 grid locations for 12,340 days. Abscissa labeled in years. (**C**) Cumulative rainfall (in mm in colorbar) on 61 × 61 grid for all 12,419 days over 34 years from 1983–2016. Map created using ArcGIS 10.4.1, http://support.esri.com/Products/Desktop/ArcGIS-desktop/arcmap/10-4-1. (**D**) Fusion of cumulative rainfall with village and country map. Note that the heaviest rains are over the rainforest within the Congo River Basin to the west of Uganda, and where the northeast corner of Lake Victoria abuts the Ugandan land mass. The driest region is in northeast Uganda, where the Karamoja district abuts northwest Kenya and South Sudan. (**E**) Spectral density using Welch’s method in decibels (dB) estimated from each of 3,721 grid locations over 12,419 days, with mean and ± 1 SD. Hamming window of 3 × 365 with 95% overlap. Points plotted as decibels (10log10). (**F**) Spectrogram with mean removed from signal. Note the dominant rainy cycles at 1× and 2× per year.

Geographically, Uganda stretches from 1.43°S to 4.27°N and from 29.5°E to 35.03°E, within a landlocked region of East Africa. At 0.1° × 0.1° resolution Uganda is contained within a square 61 × 61 grid. In Fig. 1A we illustrate the country as a composite of the boundaries of the 44,034 villages that comprise the landmass, and superimpose the 3,721 satellite grids overlay.

The entire data set for all 3,721 time series (plotted in sequential colors) is illustrated in Fig. 1B, demonstrating the complexity and range of such data. The cumulative rainfall over 34 years is mapped onto the satellite grid in Fig. 1C. Fusing the cumulative rainfall with the country map in Fig. 1D, there are several notable features. In the northeast, the semi-arid region of Karamoja is shown with low cumulative rainfall (dark blue). The heaviest rainfall is in the northwest over the Congo River basin rainforest (dark red). The other region with high rainfall is where the northeast edge of Lake Victoria meets the Ugandan landmass in the lower central region of the plot.

The averaged power spectral density of all 3,721 time series with error bounds is shown in Fig. 1E, which reflects the dominant 1- and 2-cycle per year frequencies, and the spectrogram in Fig. 1F demonstrates the consistency of these two fundamental frequencies throughout the 34-year record. The 2-cycle per year rainfalls in the East African Highlands are of unequal size, augmenting the 1-cycle per year frequency amplitude.

**Figure 2:**
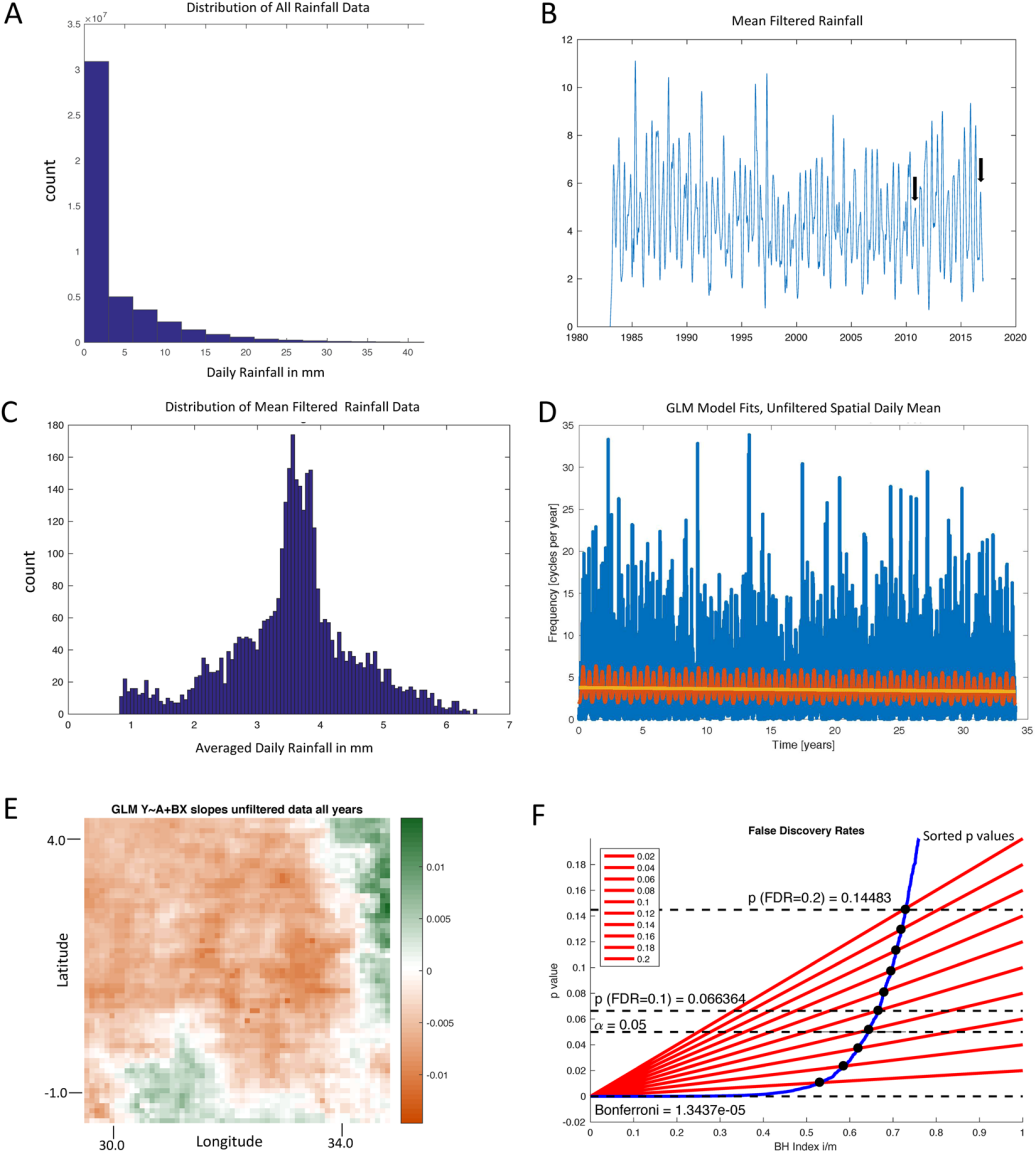
Spatially averaged rainfall demonstrates a decrease over the 34 year record. The nature of the data with and without spatial averaging is shown, and the origin of the average decrease in rainfall explained by the geographical distribution of decreases and increases in rainfall. (**A**) Histogram of daily rainfall for all days and locations, truncated below 40 mm rain per day. (**B**) Daily rainfall spatially averaged, and filtered with finite impulse response filter (order 100) for frequencies 0.05–6 cycles per year. Filter applied with zero phase filtering forwards and backwards. The twice-yearly rainy seasons in the East African Highlands are now plainly seen. Two drought anomalies from droughts in fall rainy season failures are indicated for 2010 and 2016 with black arrows. The averaged and filtered data set from B demonstrates a much less skewed distribution in (**C**). (**D**) Linear GLM fit, log(μ) = A + BT, shown by yellow line, and full model fitted with 0.25, 0.5, 1, 2, and 4 cycle per year frequencies shown by red line, superimposed on spatially averaged but unfiltered data in D. In E are shown the individual slopes of linear GLM regression to each grid point time series, with no averaging, on a scale illustrating the intensity of the negative (brown) or positive (green) slopes. Map created using ArcGIS 10.4.1, http://support.esri.com/Products/Desktop/ArcGIS-desktop/arcmap/10-4-1. (**F**) False Discovery Rate (FDR) plot for the GLM fit p-values from the slope plot in E, for a family of FDR values. The intersections of the sorted p-values (blue) with the Benjamini-Hochberg coefficients (red lines) form the FDR thresholds employed in Fig. 3.

Statistically, rainfall distributions are often non-Gaussian due to non-negativity and pronounced skewness. For such data, the normal distribution will inadequately account for the rainfall variability. This renders ordinary least squares, with the assumed normal distribution of errors, a problematic choice for model fitting. Indeed, the rainfall distribution for all 46,211,099 daily measurements is highly skewed (Fig. 2A), and it is well known that such rainfall data may follow a gamma distribution^13^. By averaging across the 3,721 spatial locations for each day in time, and then filtering between frequencies of 1/20 to 6 cycles per year to eliminate outliers for visualization, the biannual wet season cycles are now readily visualized (Fig. 2B). Furthermore, the distribution of the averaged 12,419 days of data becomes much less skewed (Fig. 2C). These data can all be appropriately fit using the generalized exponential family of distributions modeled within the Generalized Linear Model (GLM) framework that embraces such distributions ranging from gamma to normal^14^.

In Fig. 2D, we demonstrate a GLM fit to the 34 years of data, spatially averaged for each day across the spatial grid but not filtered over time. We show both a log-linked linear fit with time, log(μ) = A + BT, where μ is the expected rainfall, T is time, and A and B are model parameters, as well as a fit of both time and a linear combination of frequencies (using 4, 2, 1, 0.5, and 0.25 cycles per year). There is a statistically significant downward slope of the GLM dependency on time of the spatially averaged rainfall by 12% over the 34-year interval (slope of −0.0038 corresponding to an exp(−0.0038) = 0.38% reduction in rainfall per year, p < 1.6 × 10^−5^, slope standard error SE = 0.00088), and the quality of the fit of time and frequencies is also highly statistically significant (F vs constant model 146, p < 7 × 10^−317^).

To examine the spatial distribution of this overall decrease in rainfall, we fit 3,721 GLM models to each of the spatially-mapped grid’s time series datasets (without averaging or filtering). The origin of the average negative slope, B, for the linear model fit, log(μ) = A + BT in Fig. 2D, is based upon a distribution of slopes with a mean of −0.0031 (0.31% reduction per year), where the probability (fraction) of negative slopes is 0.78 (2,902/3,721). We now plot these individual slopes in their spatial location on the satellite grid in Fig. 2E. The color map for the spatial distribution of these slopes uses the intensity of brown and green to represent the magnitude of the decrease or increase in slope, respectively. There is a broad region in central and western Uganda that appears to be responsible for the overall decrease in rainfall shown in Fig. 2D.

**Figure 3:**
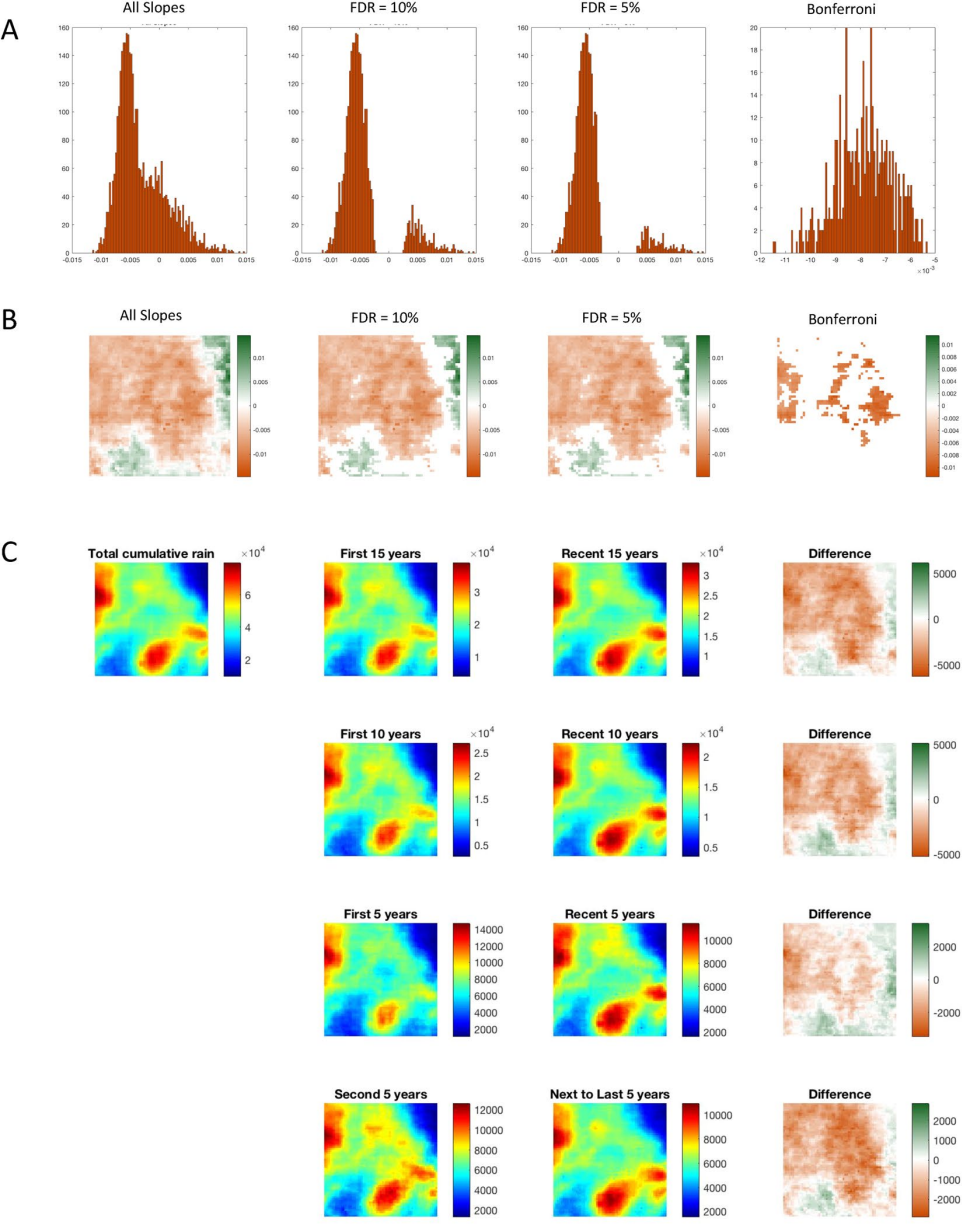
The decrease in rainfall is a robust finding. We first performed an exploration of false discovery rates, and then independently tested with cumulative rainfall differences. (**A**) Histogram of the 3,721 GLM slopes from Fig. 2E. With progressively more conservative FDR rates, we see the progressive elimination of small magnitude slopes, culminating in the most conservative thresholding with Bonferroni (α/3721). In (**B**) we map these distributions of slopes back onto the satellite grid. Note that the most significant slopes retain the coherent regions of central and western Uganda where the rainfall decreases the most over the historical record. To independently test these findings, we examine the difference maps of cumulative rainfall from the raw ARC2 data in (**C**). Indeed, for the first and most recent 15, 10, and 5 year cumulative data, and the second and second-to-last 5 year periods, the difference maps retain the same rainfall pattern of decrease indicated from the GLM fits. Maps created using ArcGIS 10.4.1, http://support.esri.com/Products/Desktop/ArcGIS-desktop/arcmap/10-4-1.

These 3,721 slopes (Fig. 2E) represent a massive multiple testing problem. To explore this spatial distribution further, we turn to the control of false discovery rate (FDR) using the method of Benjamini and Hochberg^15^. We plot the curve representing the GLM goodness of fit as p-values (in blue) in Fig. 2F against a family of FDRs ranging from 0.02 to 0.2, along with the standard FDR for a single test (0.05) and the Bonferroni corrected false positive rate of 1.3 × 10^−5^. The distributions corresponding to these FDRs are shown in Fig. 3A, and their corresponding spatial maps in Fig. 3B. As the false discovery rates vary from 0.1 to the Bonferroni rate, identification of the region of central and western Uganda with decreasing rainfall over these 34 years remains robust.

We can independently assess these decreases in rainfall by taking difference maps of cumulative rainfall for different periods of time. In Fig. 3C, we illustrate the difference of cumulative rainfall in the first (oldest) vs last (most recent) 15, 10 and 5 years of the data, along with the second 5 vs next to last 5 year segments of data. The spatial maps visualizing increases or decreases in rainfall depict a broad region of decreased rainfall within central Uganda that remains consistent with the GLM regression slopes in Fig. 3A and B.

**Figure 4:**
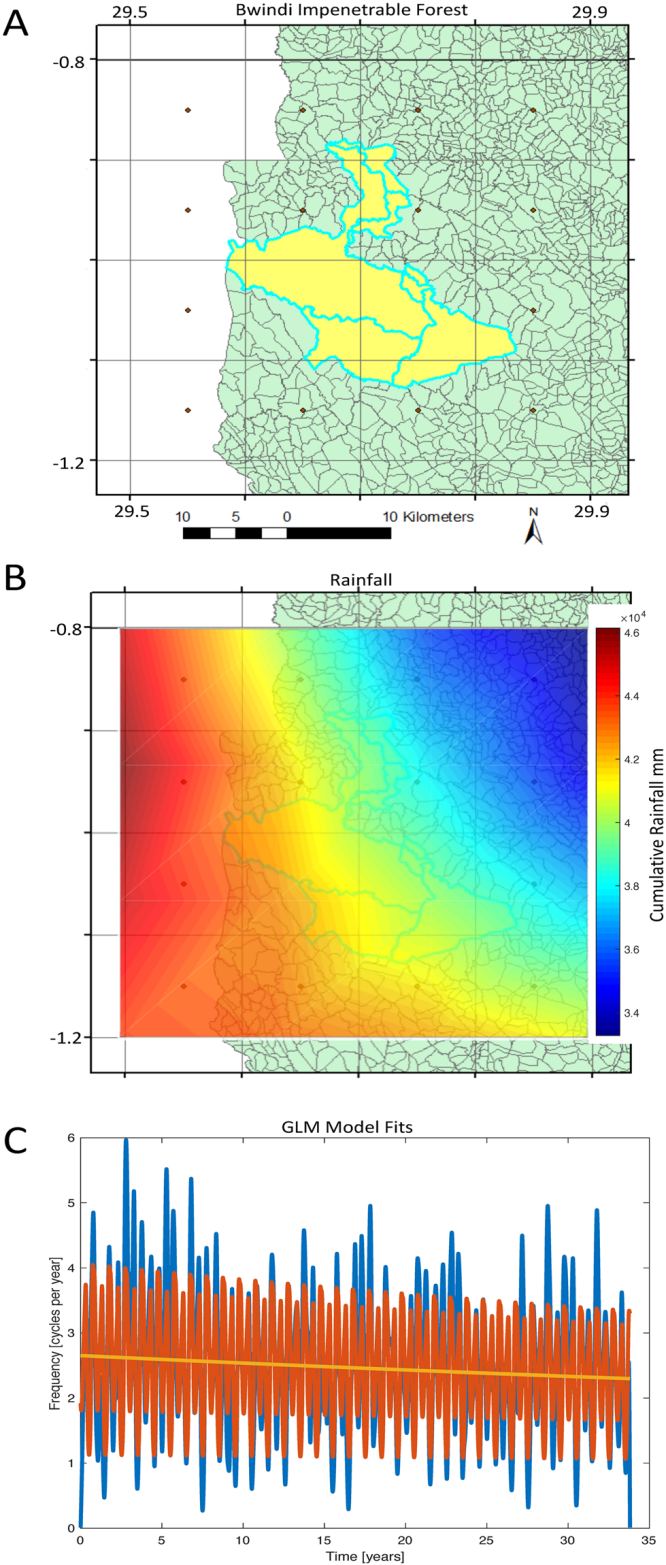
The decrease in rainfall is also reflected within the Bwindi Impenetrable Forest region. This is one of the last remaining habitats of the Mountain Gorilla. (**A**) The Bwindi Impenetrable Forest in Uganda (yellow), covered by 16 of the satellite grids (map created using ArcGIS 10.4.1). The Democratic Republic of the Congo is in white. (**B**) Cumulative rainfall from 1983–2016 with interpolated shading over the Bwindi coordinates. (**C**) Spatially averaged filtered time series of the Bwindi rainfall, overlain with the GLM models as in Fig. 2. Note the substantial rainfall decrease over the record.

Next, we examine the Bwindi Impenetrable Forest within Uganda. This forest reserve constitutes one of the last remaining habitats of the Mountain Gorilla, and is considered crucial for species survival. Ground based rainfall data are understandably incomplete because of remoteness and inaccessibility^4^. The coordinates of the 331 square km Bwindi Forest lies within 0.85°S and 1.15°S, and 29.55°E and 29.85°E on the satellite map, the southwest tip of Uganda. These coordinates correspond to 16 squares of our grid (Fig. 4A). The cumulative rainfall from ARC2 is projected on the Bwindi Forest region and shown in Fig. 4B. We again find that there is a statistically significant downward slope of the GLM dependency on time of the spatially averaged rainfall by 13% over all years (slope of −0.004 corresponding to a 0.4% decrease in rainfall per year, p < 0.012  slope SE = 0.0015), consistent with our findings in the entire national grid.

**Figure 5:**
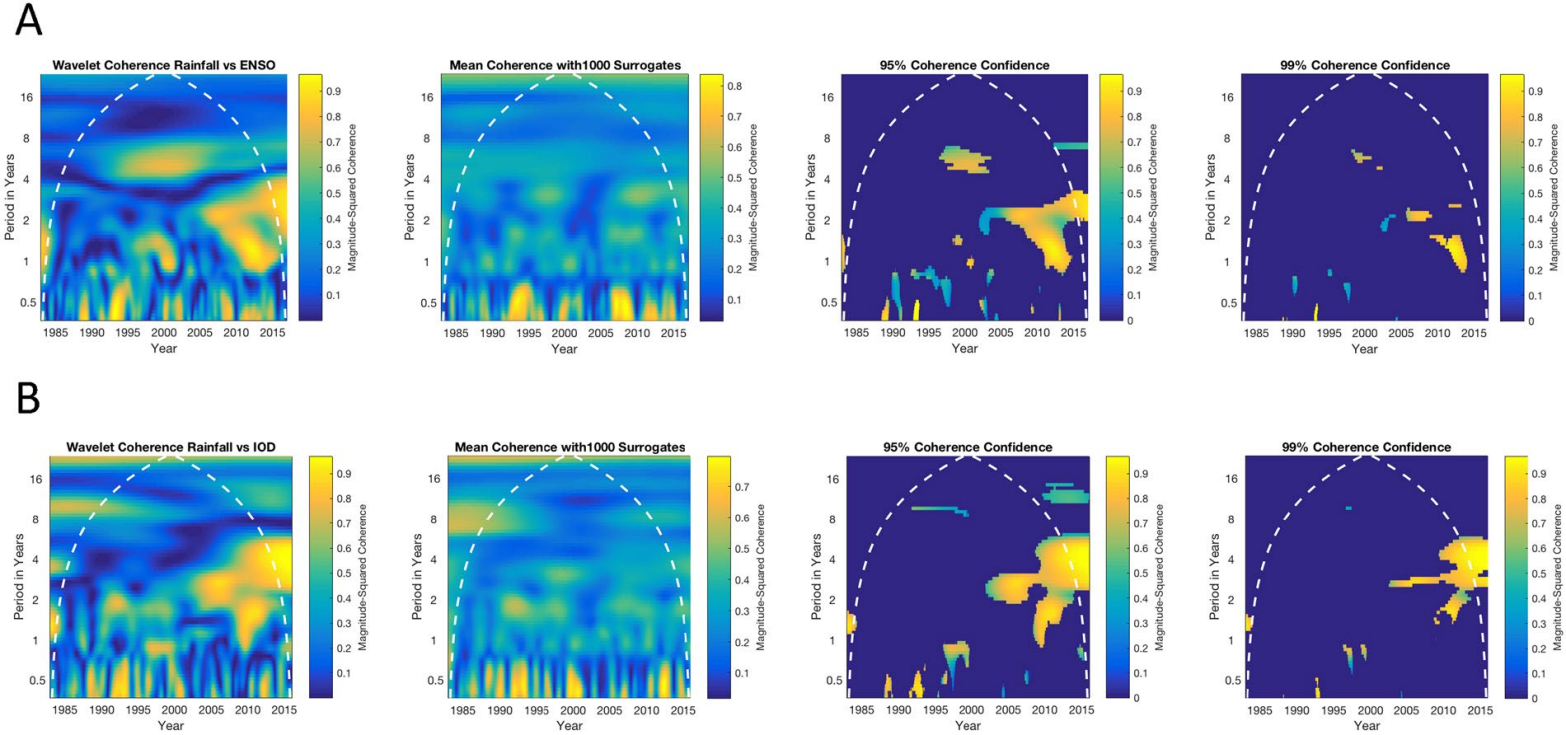
El Nino Southern Oscillation (ENSO) and Indian Ocean Dipole (IOD) relationship to spatially averaged rainfall. With rigorous statistical confidence bounds, there is a coherency between these indices and the Ugandan rainfall, but only in recent years, and only for relatively short periods from 1–4 years. (**A**) Wavelet coherency between spatially averaged and filtered rainfall, with the ENSO index interpolated from monthly to daily values, and filtered identically as rainfall data. The second panel reflects the mean of 1000 surrogate coherency calculations, from which 95% (third panel) and 99% (fourth panel) confidence limits reveal only the regions that met these significance criteria. The cone of influence, delimiting where edge effects substantially confound the analysis, is indicated by the white dotted line. (**B**) Identical calculations for the IOD index. The most significant coherencies between ENSO and IOD occurs during the most recent 10 years, with periods from 1–4 years.

Lastly, both the El Niño Southern Oscillation (ENSO) and the Indian Ocean Dipole^16^ (IOD) are known to influence rainfall in East Africa^17^. We turn to the technique of wavelet coherency^18^, and seek a statistical bootstrap that preserves more of the properties of the data than the white^19^ or colored^20^ noise employed in previous work on geophysical systems. Following the recommendation for a non-parametric bootstrap constructed from surrogate data from the original time series^18^, we used a randomization scheme previously employed by swapping binary partitions at random locations in a time series^21^, to ensure that local correlations are effectively destroyed across an ensemble of such resampled time series, and that the relevant frequencies and distribution of values remain the same. Applying this bootstrapped statistical method, our results showed there are regions at the 99% confidence limit in the latter half of our time series where ENSO (Fig. 5A, 2006–2014, 1–2 year periods) and IOD (Fig. 5B, 2004–2015, 1–4 year periods) coherency with rainfall remains significant.

The relatively short length of the instrumented ARC2 limits our analysis to 34 years. Proxy and model simulation suggests that the IOD is more important than ENSO over multidecadal and perhaps longer time scales^22^. Although ENSO effects interannual droughts^22^, it is less important for interdecadal rainfall patterns^23^, and our results are consistent with this. Nevertheless, our findings that ENSO and IOD effects on Ugandan rainfall have been more significant within the more recent half of the 34-year record remains unexplained.

The weather patterns in East Africa are unusually complex and regionally disparate^23,24^. Uganda is at the western edge of the Greater Horn of Africa^25^ region, and has historically had more rainfall than neighboring Kenya and Tanzania^26^. One of the hazards in generalizing from regional models of the Greater Horn of Africa to more localized regions, or extrapolating using proxy data taken from highly localized lake or ocean sediment core analysis^23,24^, is that predictions do not equally apply to all countries within such regions. There is a well-known discrepancy between coastal Horn of Africa rainfall and more interior Rift Valley sites^23^. In our present analysis, the multi-decadal influence on East African rainfall predicted from IOD dynamics is not well reflected in our 34-year Ugandan dataset, where the effects appear sub-decadal.

The biannual rainy seasons in East Africa are driven by different dynamics, with the climate circulation and sea surface temperature affecting each type of rainy season differently^22^. Over the past 60 years, the Indian Ocean has warmed more than the tropical Pacific Ocean, which may account for a westward extension of the tropical Walker circulation, which is in contrast with models that predict a weakening of the Walker circulation^27^. Such an effect would account for weakening of the long rains (March – June), and would potentially be independent of ENSO effects. Although the Horn of Africa region has become observationally drier during the 20^th^ century, many climate models predict an increase in short rains (September – November) as global temperatures rise^23,24^. The current discrepancies between the proxy sediment record and 20^th^ century observations, with model predictions predicting an increase in rainfall over the eastern Horn of Africa with global warming^24^, are indications that more accurate model simulations and an improved understanding of the geophysical processes governing the rainfall over East Africa are needed given the fragile food security issues of this region.

How such changes in rainfall patterns impact infectious disease prevalence and risks will be determined by individual disease characteristics, and will be important for specific locations. By fusing the satellite rainfall grid with the locations of all of the villages in Uganda, we have a finely granular way to track epidemic diseases given village case data for individuals who become ill. By helping to identify vulnerable locations, such fusion functions as a platform for seeking optimization of treatment and prevention of many infectious diseases, of which we are particularly focused on neonatal sepsis^28,29^ and a critical sequela in survivors in Africa, postinfectious hydrocephalus^10–12^.

Although climate is global and regional, policy and preparation remains largely dependent upon individual countries, where the consequences of climate and remediative responses are complexly related to local conditions. Uganda is a country where 72% of geographical area is used for rain-fed farming and the population growth is one of the highest in the world^4^. An average rainfall decrease of the magnitude reported here, over the multiple decades of the climate record examined, is important for sustainable agricultural decisions in a country dependent on subsistence crop yields. Additionally, understanding the fine-scale spatial vulnerability of such rainfall declines can help generate more efficient decision-making and resource use allocation. There is a substantial need for more granular and accurate prediction modeling for both short-term drought anticipation and longer-term rainfall trends within the time-frame relevant for economic planning. Nevertheless, the present trend in rainfall decrease is gradual enough so that there remains an opportunity to build adaptive capacity^1^ through strategies^25^ to make the country more resilient: anticipatory land-use management, shifts towards more sustainable agricultural practices, wastewater reuse, and infrastructure development to increase the resiliency of the society with respect to short and long-term changes in rainfall.

## Methods

### Data

Rainfall data was obtained from the African Rainfall Climatology, version 2 (ARC2)^3^, the gridded daily 34-year precipitation estimation dataset (http://www.cpc.noaa.gov/products/international/data.shtml) centered over Africa at 0.1° × 0.1° spatial resolution. These data are an estimation derived from a fusion of the geostationary infrared sensing from the European Organisation for the Exploitation of Meteorological Satellites, and 24-hour rainfall measurements from Global Telecommunication System gauge observations. There are 341 missing days in these ARC2 data (out of 12,419 days) which were accounted for by linear interpolation.

It is important to note that the estimation of rainfall from satellite data suffers from uncertainty and bias, which varies with geographical topography. The ARC2 data accuracy has been compared with ground station data along the Albertine Rift in Western Uganda. It tends to overestimate rainfall days, biased within a range of −17% to −12% along northern sections of this region, but with minimal bias in the Bwindi region^4^. On the other hand, ARC2 has fewer false alarm rain days than the more recent products such as Rainfall Estimator (RFE) data (mean false alarm ratio 0.41 range 0.29–0.60, vs mean 0.44 range 0.33–0.61)^4^. In addition to the satellite infrared and ground based gauge data from ARC2, RFE adds microwave sensing, and 3B42v7 has finer temporal and spatial resolution along with a more advanced algorithm^4^. Nevertheless, in validating each of these data against ground station data along the Albertine Rift, there are biases and errors for specific sites that render each of them problematic if applied without ground based station data. ARC2 is the only method that extends earlier than the year 2000, and appears a reasonable choice to apply to gauge-deficient regions of central Africa^4^ as we have here done.

El Nino Southern Oscillation data (ENSO) was obtained from the Multivariate ENSO Intex (MEI) from the Earth System Research Laboratory at NOAA at https://www.esrl.noaa.gov/psd/enso/mei/table.html. These monthly data from 1983 through 2016 were then uniformly expanded across the number of days in each month to obtain equivalent daily data.

The Indian Ocean Dipole (IOD) data was obtained from The Extended Reconstructed Sea Surface Temperature (ERSST) dataset derived from NOAA’s International Comprehensive Ocean-Atmosphere Dataset (ICOADS), accessed at https://iridl.ldeo.columbia.edu/SOURCES/.NOAA/.NCDC/.ERSST/.version4/.IOD/.C1961-2015/.iod/datatables.html. Similar to the ENSO data, these monthly data from 1983 through 2015 were then uniformly expanded across the number of days in each month to obtain equivalent daily data.

### Generating Maps and Grids

#### Creating Maps of Uganda at the Village Level

The geocoded data of Ugandan village boundaries was obtained through the Ugandan National Planning Authority, by compiling a shapefile using census and election commission records, accessible at [https://scholarsphere.psu.edu/concern/generic_works/k0p096889c]. The Uganda shapefile was entered in ArcGIS version 10.4.1 for desktops (Environmental Systems Research Institute (ESRI), USA, http://support.esri.com/Products/Desktop/ArcGIS-desktop/arcmap/10-4-1) and converted to points using the ArcGIS ‘feature to point’ tool. Then, the centroid of each village was used to generate the XY coordinates with the ‘geometry’ tool.

#### Satellite Grid Registration

The ‘fishnet’ tool in the data management toolbox of ArcGIS was used to create the grid at 0.1° × 0.1° (Fig. 1A). The centroid of each grid square was used to generate the XY coordinates with a ‘calculate geometry’ tool. Grid point (1, 1) was the latitude 1.9°S, longitude 29.5°E. The grids increment by 0.1° until the extent of Uganda is enclosed (1.9°S–4.2°N and 29.5°E to 35°E).

#### Bwindi Impenetrable Forest Map

Bwindi impenetrable forest (Fig. 4A) was selected from the Ugandan map using the ‘select by attribute’ tool in ArcGIS. The selected features were then saved as a shapefile using the ArcGIS ‘create a layer from selected feature’ tool.

### Signal Processing

Spectra were calculated, following removal of the mean from each of the 3,721 time series, using Hamming data windows 3-years in length (3*365 days), with 95% overlap of these windows, and 1-sided spectral estimations performed using Welch’s method as implemented in Matlab function *pwelch*^30^. The power spectral density (PSD) from each of the 3,721 time series from the different locations were then averaged, and plotted on a decibel scale (10*log(PSD)) in Fig. 1E for frequencies greater than zero and less than 12 cycles per year. The spectra from each windowed time period were then assembled into the spectrogram in Fig. 1F. When filtering was applied, such as in Fig. 2B, we employed a bandwidth of 1/20 to 6 cycles per year, an order of 100, frequency of 365 days per year, Nyquist frequency of 365/2, and a finite impulse response (FIR) filter (Matlab function *fir1*) applied in a zero-phase distortion manner using Matlab function *filtfilt*. The spatial mean of all 3,721 filtered time series were plotted in Fig. 2C. When employed in wavelet coherency, both the spatially averaged rainfall time series (through 2015), and ENSO time series were equivalently filtered within the same 1/20 – 6 cycle per year bandwidth.

### Statistical Analysis

#### Generalized Linear Modeling

We implemented generalized linear modeling (GLM) using the methods developed by Nelder and Wedderburn^14,31^. Using the implementation of the Matlab function *glmfit*, we employed gamma distributions and log link functions.

#### False Discovery Rate

The false discovery rate (FDR) was controlled using the method of Benjamini and Hochberg^15^. A family of FDR rates was developed, assuming that the false positive (Type I) error rate was α (nominally 0.05), the number of comparisons tested was *m*, as the largest *p(i)*, where:

$$p(i)\le \alpha \ast \frac{i}{m},0\le i\le m.$$

These *p(i)* thresholds, for a family of FDR rates, are shown as intersections between the blue and red lines in Fig. 2F.

### Wavelet coherence

We employed the method of Torrence and Webster^19^, as implemented in the Matlab function *wcoherence*, except we replace their white noise surrogate with surrogates based upon randomly partitioned surrogates^21^ using each one of the time series. We chose the boundaries of the partitioned sections randomly, swapping the partition order. This has proven a robust method to break up short-term correlations between two time series, while preserving the statistical properties other than losing the slowest of frequencies due to the partitioning. Unlike preserving spectra by randomizing the phases of frequencies in a Fourier transform, and then inverting the transform, this method also preserves the original data values and distribution^21^, and is much more computationally efficient than simulated annealing alternatives^32^. We created an ensemble of 1000 such surrogate wavelet coherencies, chose the largest 95% or 99% surrogate coherencies at each point in the time-frequency plot, and used these thresholds to build our bootstrapped confidence limits setting all values to zero which did not exceed these thresholds. We plot the remaining significant wavelet magnitude-squared coherencies in the significance plots of Fig. 5.

### Code availability

The data sets, and Matlab code required to replicate the findings of this papers, are openly available at [https://scholarsphere.psu.edu/concern/generic_works/k0p096889c].
